# Exploring motivations for participating in research among Australian women with advanced gynaecological cancer: a qualitative study

**DOI:** 10.1007/s00520-023-07979-x

**Published:** 2023-08-08

**Authors:** B Wigginton, M M Reeves, T DiSipio

**Affiliations:** School of Public Health, The University of Queensland, 288 Herston Road, Brisbane, Queensland 4006 Australia

**Keywords:** Gynaecological cancer, Metastasis, Advanced cancer, Qualitative, Australia

## Abstract

**Purpose:**

With global moves to increase research among those living with advanced cancer and legitimise consumers as part of cancer research, this article aims to build an understanding of women’s motivations and reasons for participating in gynaecological cancer research. As a secondary aim, we considered the role of qualitative methods in enabling active involvement of consumers in research.

**Methods:**

We applied thematic discursive analysis to 18 in-depth interviews with women diagnosed with advanced (stage III–IV) gynaecological cancer living in Australia.

**Results:**

We found that women viewed *research as a vehicle for change* in two directions: improving the lives of future generations and improving education and awareness. Underpinning these two framings of research, women spoke about their own role and reasons for participating in this interview study. Women’s stories were painted against a backdrop of social and medical *silences around gynaecological cancer*. It was from such silence that women chose to speak up and position themselves as *participating in service for knowledge production.*

**Conclusion:**

We learned that trust, reciprocity and relationships are central to women’s decisions to participate in cancer research. Legitimising consumers in cancer research requires methods, methodologies and practices that pay careful attention to power, control and representation.

**Supplementary Information:**

The online version contains supplementary material available at 10.1007/s00520-023-07979-x.

## Introduction

Gynaecological cancers start in the female reproductive system or genitals and include cervical, ovarian, uterine, vaginal and vulvar cancer. In Australia, the number of women diagnosed with gynaecological cancer has substantially increased over the past two decades (3802 in 2001 to 6576 in 2021) [1]. This accounts for almost 1 in 10 female cancer cases. While 5-year survival rates are relatively high for those diagnosed early, they significantly reduce with increasing stage at diagnosis [2]. This has significant implications given 1 in 3 women diagnosed with gynaecological cancer is diagnosed with advanced disease; this is largely driven by the higher proportion (~70%) of ovarian cancer diagnosed at stage III–IV [2]. While qualitative evidence documenting psychosocial experiences and impacts of diagnosis and treatment of gynaecological cancer is emerging e.g. [3–6], what is less well understood is an understanding of women living with advanced gynaecological cancer as well as their interests and visions for cancer research. A seminal meeting held by the National Cancer Institute in 2019 identified the need to better understand cancer survivorship for those living with advanced cancers [7], and a subsequent meeting held in 2021 focused on this priority area [8]. However, researching advanced cancer is challenging because of the additional burden of the disease itself, such as deterioration in health, feeling distressed, symptom burden, physician refusal and death [9, 10]. Yet these complexities make research with advanced cancer populations even more important, especially in light of global calls to involve consumers^1^ in health research [11].

Consumer involvement can involve diverse activities from consultation to co-design and co-production in a deeply collaborative sense [12]. Global acknowledgment is evidenced by funding bodies requiring that proposals involve consumers [13]; for example, in the UK, the National Institute for Health Research and the National Health Service and in Australia, the Medical Research Future Fund and Cancer Australia grant schemes. With such shifts, evaluating the impact of consumer involvement has become an important area of research [14, 15], as well as the development of frameworks to facilitate consumer involvement in research activities [16]. For example, Cancer Australia in partnership with Cancer Voices Australia published a National Framework for Consumer Involvement in Cancer Control intended to ‘support organisations committed to involving consumers in cancer control’ and to ‘facilitate consistent approaches to consumer engagement’ (p. 1). This framework is advocated on the assumption that when consumers are integral and equal members of research, health services and policy development, there are improved outcomes and experiences for those affected by cancer—an argument also reflected in the literature [16].

This article aims to build on these moves to legitimise consumers as part of the research process, as well as better understanding the lived experiences of advanced cancer populations. Qualitative methods have received much attention in terms of the opportunities to inform and evaluate randomised trials of complex healthcare interventions see reviews: [17, 18], improve informed consent processes [19] and design epidemiological studies [20]. While qualitative research can improve the feasibility and outcomes of such research, it has also been argued for on moral and ethical grounds [13]. This begs the question, why do consumers participate in research and especially people living with an advanced diagnosis? We argue that understanding consumer motivations and reasons for participating in research is an important question if we are to further consumer engagement in cancer control research.

For the global and national calls to *meaningfully* involve consumers into the research activities and involve them in shaping agendas and activities, we query whether qualitative research offers unique methodological grounds to do this work. In that vein, this article explores the value of a qualitative approach to support consumer involvement in the design of the gynaecological cancer research agenda and activities in the context of an interview study based in Australia (a secondary aim of this study).

In our study, we did not require women to have had any prior experience with research to share their interests and visions for cancer research. Drawing on in-depth interviews, we explored Australian women’s perspectives around participating in research *in relation to* their experiences of being diagnosed with advanced (stage III–IV) gynaecological cancer. As a secondary aim, we were interested in the role of qualitative methods in enabling active involvement of consumers in research.

## Methods

Ethics approval was obtained from The University of Queensland Human Research Ethics Committee (HE000877). The COREQ checklist for reporting qualitative research was followed [21]. The study was advertised through a national women’s cancer charity, Cherish Women’s Cancer Foundation (as approved by the Foundation Board) through their newsletter and social media pages, including a link to the participant information sheet and consent form. Anyone who received the advertisement was invited to distribute the information to others as a snowballing recruitment approach. Women were eligible if they were diagnosed with advanced (stage III–IV) gynaecological cancer, aged 18 years or older, lived in Australia and spoke English. There were no requirements around prior experience participating in research. Women interested in participating in the study provided their contact details to the research team via a secure online form. Women were telephoned by a member of the team to provide further explanation of the study, screen for eligibility and answer any questions. Verbal consent was obtained from those willing to participate, and a suitable time for the interview was scheduled. Participants completed and returned a signed consent form prior to their scheduled interview. Women received a $25 gift voucher as an appreciation of their time for participating. Recruitment continued until the authors agreed that there were no new themes emerging from interviews (i.e. data saturation).

Twenty women expressed interest to participate in the study. Of these, two women were not eligible: one was not diagnosed with stage III/IV disease and one was a caregiver. A total of 18 women were eligible and subsequently interviewed between September 2021 and May 2022 via telephone by BW at a time and day convenient to participants. She had no prior interaction with the participants nor involved in their care. Interviews lasted between 40 min and 2 h (an average of 1 h). Interviews were audio recorded and transcribed verbatim by a professional transcription service.

The interviews were underpinned by a feminist methodology [22] in which gender and intersecting structures (race, class, sexuality, etc.) are foregrounded in the research alongside the practice of reflexivity. In this vein, we acknowledge that our team includes public health researchers from various career stages. The lead author is trained in psychology and is an experienced qualitative researcher. All members of the team (BW, MR, TD) have a family history of cancer.

A feminist methodology supports a participant-directed approach in which interview questions are moulded to women’s language use, enabling free-flowing conversation rather than directing general questions in a linear fashion. This methodology also supports an awareness of, and attention to, power in the interview, as well as the emotional dimensions of participants’ experiences [22]. A semi-structured interviewing style [22] enables active listening, silent probes, minimal encourages and a host of other techniques to draw out and hold complexity within a story—especially important in an advanced cancer context. All interviews covered the following topics: diagnosis and treatment; the role of research; interest in, and preferences around, participating in research; advice for women in a similar situation (Online Resource 1). No formal pilot interviews were conducted—rather the research team developed the interview guide together, led by TD, with iterations of feedback to ensure its applicability and appropriateness to the participant group.

### Analysis

Thematic analysis is a theoretically flexible method that enables attention to description and interpretation—going beyond the content to consider underlying meanings and implications [23]. We were particularly interested in women’s conceptualisations^2^ of ‘research’, including their aspirations and their role as participants. One researcher (BW) led an inductive analysis of the interviews guided by this interest in conceptualisations of research. The interviewer and analyst (BW) has a PhD in health psychology, with over a decade of experience conducting qualitative research, primarily interviews, in women’s health and sensitive health topics, including advanced cancers. Her training in feminist methodologies also emphasises the role of reflection and critical analysis of herself within the research.

BW started with a process of familiarising herself with the data (reading and rereading transcripts and fieldnotes) followed by manual descriptive coding to organise potential data patterns and relationships. Descriptive coding began with coding each individual interview for points of interest (e.g. why they chose to participate in this research). BW looked beyond stated reasons for participating, to also observe *how* women participated within the interviews. For example, some women initiated a discussion about this being their first time participating in research and offered a candid account about why they chose to be interviewed. This pivot was analytically interesting, including *who* initiated the conversation about research and *how* and allowed BW to bring a discursive thread [24] to the analysis in order to provide a detailed account of how women positioned themselves within the interview and what emerged (i.e. the three overarching themes) as well as the potential lessons this perspective might offer for cancer research: the secondary aim.

Descriptive codes were shared with the other two authors who provided feedback. BW integrated this feedback as she developed a conceptual map of the patterns across the data. This process involved collating descriptive codes across interviews and considering their interconnections and how multiple codes clustered together to form a theme. She defined themes as a patterned response or meaning at some level in the data [23]. BW identified three overarching themes: *the silence of gynaecological cancers*, *research as a vehicle for change and participation as service for knowledge production*. These will be explored here to provide sufficient context to gynaecological cancer experiences and women’s participation in this research.

The secondary aim emerged not only from the content of the findings but also through BW’s reflexive process during data collection, where she reflected on the influence of the methodology and methods on the stories women were able/willing to share (i.e. the data). We share these as *lessons* to highlight process learnings to support future research in this area.

Themes were shared with participants in a brief report, who were then invited to provide feedback or comment; none of whom chose to do so. Participants were also invited to join a consumer reference group to inform the future direction of the research; to date, four have joined the group and have provided input into research activities.

## Results

Participants ranged in age from 31 to 74 years (median 57.5 years) from across Australia (61.1% regional/remote) (Table 1). The average time since diagnosis of advanced cancer was 21.5 months (range 1 to 149 months) at the time of the interview.

**Table 1:** Demographic and clinical characteristics of study participants (*n* = 18)

Characteristics	*N* (%)
Age, median (minimum, maximum)	57.5 (31, 74)
Months since diagnosis of advanced cancer, median (minimum, maximum)	21.5 (1, 149)
Treating hospital	
Public	5 (27.8)
Private	13 (72.2)
Highest education level	
Grade 10	6 (33.2)
Grade 12	3 (16.7)
Trade/certificate/diploma	3 (16.7)
Undergraduate degree	3 (16.7)
Postgraduate degree	3 (16.7)
Residential location	
Major city	7 (38.9)
Regional/remote	11 (61.1)
Gynaecological cancer	
Uterine/endometrial	7 (38.9)
Ovarian	5 (27.8)
Cervical	2 (11.1)
Vaginal	1 (5.5)
Vulval	3 (16.7)

Through the interviews, we found that *research was conceptualised as a vehicle for change* in (1) improving the lives of future generations of women through improved diagnostic and treatment pathways and (2) improving public awareness and education around gynaecological cancers, particularly addressing the *silence* and associated knowledge gaps in the general public and within the medical/health profession. Women’s stories revealed much of the wider socio-political context in which women’s diagnoses and treatment experiences were taking place. We begin by exploring this wider context in which women’s participation in research, as *service for knowledge production,* is situated.

### The silence of gynaecological cancers

Women consistently described the invisibility of, and silence around, gynaecological cancers. Many women described how their personal networks (e.g. family, friends) and some of the healthcare professionals they were interacting with (e.g. General Practitioners (GPs), nurses) had little awareness or education about gynaecological cancers:

“I just don’t think that enough attention is given to ovarian cancer. You know, you walk into a doctor’s surgery and, and the, the information regarding breast cancer hits you in the face just about as you walk, walk into the room. That, that’s not the case with ovarian cancer. And I particularly had, had no idea that I was a candidate for ovarian cancer.” (P115)

P115 repeatedly describes herself as “lucky”^3^ that her young female GP acted quickly in testing her symptoms that the participant had dismissed as a urinary tract infection. We share P115’s account because it is against a backdrop of invisibility around gynaecological cancers; women articulated their gratitude for a quick diagnosis from a “switched on” GP, as P115 described her. However, many women described the lengths they had to go to in order to get diagnosed. Some described male GPs who “fobbed me off” (e.g. P101); others described their symptoms dismissed or minimised by GPs (e.g. P110, P114) or delays to their diagnosis due to COVID-19 restrictions and impacts on the healthcare system (e.g. P103).

Two participants, both diagnosed with vulval cancer, had, in their own words, “traumatic” experiences of treatment and side effects from surgery that led to dissociation from their body (P108) or intense post-traumatic stress (P109). For example, P109’s diagnosis pathway was significantly delayed despite having been at increased risk of cancer having lived with lichen sclerosus since she was a child; she struggled to find a doctor to look “seriously” at her vulval lump:

“I went to two doctors, one wouldn’t even look at me and just gave me some strong antibiotics and said “you’ve probably got an infection. Um, there you go”. Another doctor stood at the end of the bed and wouldn’t touch me or have a look. He just sort of like, you know, pretended to sort of look but wouldn’t. Um, and over a period of eight months I went to five different doctors. I was admitted to ED [emergency department] three times because I couldn’t walk around in that much pain, and um each time I got tested for an STD [sexually transmitted disease] because of my age group… so it took a whole year from when I first noticed the lumps to diagnosis.” (P109, diagnosed at 26 years old)

The significant social and emotional impacts of treatment and the associated side effects that were often described as “horrible”, “shocking” and “traumatic” were not exclusive to women diagnosed with vulval cancer—it was evident across all gynaecological cancers.

We also noted across the interviews the taboo nature of gynaecological cancers. For instance, women acknowledged the shame around the cancer type (e.g. P109, P110, P115) or experiencing judgement for being diagnosed with a gynaecological cancer (P114). One participant described a “horrible” experience of being admitted to hospital for 10 days and hearing a trainee nurse ask another nurse “how do you get vaginal cancer”:

“and, and, and the other nurse said, “oh she’s been spreading the love around.” Right at the door.” (P114)

This silence, misunderstanding and isolation women described were also in part feeling isolated from a system that does not ‘understand’ the cancer type—particularly noticeable in younger women’s accounts of having a post-menopausal cancer (e.g. P115). The societal silence was mirrored by the silence of these cancers, with little or easily misplaced symptoms. As one participant said, “it [ovarian cancer] is the silent killer. It can kill you without you even realising what’s going on” (P103).

### Research as a vehicle for change

The second theme conceptualised *research as a vehicle for change* in two material ways: improving the lives of future generations and improving education and awareness (see summary in Table 2). As a ‘vehicle for change’, the sum impact of research was seen to be potentially greater than the sum of the parts: “without research, nothing advances” (P113).

**Table 2:** Summary of women’s vision for gynaecological cancer research

Directions for improving health care systems (diagnosis and treatment)	Directions for improving public awareness and education around gynaecological cancers
Reducing the cost of treatment and increasing transparency around costs	Promote screening for all gynaecological cancers
Ensuring equitable health care across public and private systems	Comprehensive information provision in schools, universities, health centres and tertiary clinics (e.g., hospitals)
Improved Medicare (government) rebates for after care support	Promote appropriate and clear language for female anatomy across settings
Trauma-informed, holistic support and care (particularly in post-operative/treatment period), including psycho-social support and sexual health support	Increase education around the surgical risks and psycho-social impacts of treatment (e.g. sexual dysfunction and well-being)

One participant put it simply, “without the research there’s no progress in the care of women in the future” (P115). We learned that research was seen as a form of ‘progress’ more broadly, and it was within this that women framed their participation.

### Participation as service for knowledge production

We noted that underpinning women’s interviews was the third theme of *participation as service for knowledge production*. While some women articulated their participation along the lines of advocacy (“you’re my voice”: P111), many did not use that language explicitly and instead spoke to research as a vehicle for change (see Table 2) in a wider context where knowledge about gynaecological cancer is lacking at best and inappropriate at worst^4^. We noted that women’s own fluency and experience in research shaped how they spoke and the extent to which they offered detailed feedback and input around future research. However, it seemed that regardless of prior experience in research or educational background, women understood research to broadly be about ‘helping’, as one participant, who had not previously been involved in research before, put it:

“Oh, um, [surgeon’s name] just rang me and said, “would you be interested [in this research]”? And I'm like, “okay. You know? If I can help someone else, why not?” I mean I- I don't know how how I could help anyone else, since I haven't got a lot of experience with it, but, yeah, may as well help out if I can.” (P118)

Some women were able to connect this altruistic notion of service to their own livelihoods/careers, such as P107 who enthusiastically recounted her surgeon inviting her into this research, to which she replied, “It’s right up my alley”, to which I queried further and she explained, “I’m a storyteller. And because word of mouth is really important to me”. Some described education and awareness raising as central to them (e.g. P108 is a teacher) and hence, their reason for participating, while others participated to be helpful (e.g. “if it helps, I’ll help”: P117).

Some women approached the interview as an opportunity to offer confidential feedback that would be received and acted upon (e.g. P108, P110; P114, P115). Implicit in this was a trust for the audience of such feedback, with some women speaking directly to this, acknowledging the legitimacy of a university conducting this research. Women’s ‘feedback’ included stories of having “slipped through the cracks” (P114) of health systems and process, as well as an absence of person-centred care. We offer two brief examples to illustrate this.

One participant with vaginal cancer (P114) described how she had been consistently told she was on a “curative treatment plan” until reading the results of a scan on her jaw, which stated “4B palliative”. No one had told her that her cancer had been restaged, and she was now considered palliative. Throughout her interview, she repeatedly stated “I’ve slipped through the cracks”, and this is why she wanted to be interviewed.

We learned from another participant (P110) with endometrial cancer that when she asked her cancer team directly what stage her cancer was, she noticed that “suddenly [they] had something important, you know, to look at [laughs] on their clipboards. Because none of them could look at me. [laughs] I said ‘Oh, it must be really bad’”.

### Lessons for future cancer research

Drawing on women’s motivations for participating in research, we also report on our key process lessons (Table 3). We learned of the importance of language use in research—especially contexts where research is attempting to give participants space to voice marginalised issues that are not adequately represented in the wider context. While some women expressed particular feedback regarding certain terms, such as “advanced cancer” which might “put people in a tailspin” of anxiety or panic (P111), others noted merely retelling the story of diagnosis as difficult. For example, one participant with ovarian cancer spoke of the difficulty of talking about her diagnosis:

“talking about my diagnosis is always hard because it's very difficult for me to put myself back in that space that I was in at the time. Um, because, because it was so painful and it was so difficult.” (P103)

**Table 3:** Lessons for recruitment

Local champions can legitimise the research	Telling the story behind the research	Research methods and methodologies shape the findings
A clinician who is leading the way in treatment and research offered legitimacy to the research and also enabled a sense of reciprocity for women (being able to return the favour to their clinician who has “saved” their life: e.g. P103)	Senior author shared her family story and connection with the research which humanised the research and shared the vulnerability of speaking about gynaecological cancer	Embedding a feminist methodology allowed us to prioritise reflections on power, language use and interview dynamics—legitimising attention to the research process (and relationship with participant) as much as interview content
Advocates who have been leading change within community through their own personal experience of cancer shared via their personal networks and recruited women with rare cancer types	Support via leading charity to promote the study in their social media platforms. This diversified recruitment and also legitimised the study through a narrative approach	Our methodology allowed the interview guide to be adapted ‘in-situ’ for each interview—not taking a ‘one size fits all’ approach to interviews. Women reported feeling “listened to and taken seriously” (P107) and appreciated being able to verbalise their experiences (P103)

We learned that participating in research requires emotional and cognitive capacities that cannot be understated. Indeed, some women told us that they could not have participated closer to their diagnosis or treatment. As P103 makes us aware, the emotional nature of the topic is not to be overlooked and was part of why only 12 months post-diagnosis, and having finished her treatment, she felt she was in “a psychological condition, sufficiently sound, to deal with it and to talk about it”.

More broadly, and implicit in our three lessons (Table 3), is the relational aspect of this research. We learned of the importance of reciprocity and trust particularly around the person who suggested them for the research, but also within the interview. We found that often the person who recruited them for research was someone they greatly trusted and respected—a leader in their community, either as a clinician or as an advocate. Some women also spoke of the authority and trustworthiness of a university, as being a reason to agree to participate—trusting the value of the research that the university produces. Again, this ties into the notion of being of *service to knowledge production* and that it matters to whom knowledge is tied to (i.e. the institution). The notion of trust was also evident within the interview itself, where women described feeling listened to and taken seriously (P107). Our methodology supported a participant-directed approach to the interviews [22] as well as care for all parts of women’s story, beyond our own research agendas, which we saw as central to enabling trust to develop in this research.

## Discussion

In-depth interviews with women diagnosed with advanced (stage III–IV) gynaecological cancer aimed to understand their motivations and priorities for future cancer research. Women viewed *research as a vehicle for change* in two ways: improving the lives of future generations and improving education and awareness. Women’s stories were painted against a backdrop of social and medical *silences around gynaecological cancers*. It was from such silence that women positioned themselves as *participating in service for knowledge production.*

Australia’s National Framework for Consumer Involvement in Cancer Control describes five types of consumer involvement, two of which we identified in our interviews: ‘personal engagement’ and ‘the advocate’ [25]. The former refers to consumers who share their personal experiences and stories as a means of raising awareness and offering feedback to health services—arguably most common in our study. The advocate represents a range of experiences and broad views of people affected by cancer, not only their own. While we saw that many participants positioned themselves and their stories from the role of ‘personal engagement’, the women who had vulval cancer offered much more of an advocacy approach in their interviews. This was evidenced by drawing on broader health system failures and gaps, the stories of other women diagnosed with vulval cancer. By contrast, several women with other types of cancer did not know any other women diagnosed with the same cancer. From our perspective, implicit in the other three types of consumer involvement (partner, expert, adviser) are nuances of health/research literacy. We observed such nuances in our interviews as influencing women’s capacity to offer detailed accounts of their visions for, and motivations around participating in, research.

With little evidence in why people participate in cancer research specifically, we briefly review evidence across diverse research topics and contexts. This literature suggests that people participate in health research for a range of reasons. Similar to our findings, these include to help others and contribute to scientific knowledge and practice [26], a belief in the importance of research [27], trusting the research institution [20], personal interests [28] or benefits [29] including access to health services [30], a belief that the research has a low perceived burden on daily life [31], because they have been asked to participate [27]. The nature and type of research is important to acknowledge as this shapes peoples’ motivations for participating, given a one-off survey is very different to a clinical trial. To date, much of this literature is based on participating in longitudinal research, clinical trials and health interventions.

A secondary aim of this study was to consider the extent to which qualitative research enacted within a feminist methodology [22] and enabled active involvement of consumers in research. Of relevance, previous interview studies have suggested that people participate in qualitative research because it is seen as innocuous or relatively unproblematic [32] and that interviews in particular offer therapeutic benefits [32, 33], providing participants with a ‘listening ear’ [34]. Our methodology foregrounded the relationship between the researcher and participant via attention to the language use in all materials, interview questions and findings shared with participants. Positioning participants in ways that afforded more control in the interview could be as simple as us asking participants to “begin wherever makes sense for you” and following their lead in the storytelling process. The emotional nature of talking about gynaecological cancer cannot be understated—again, a feminist methodology underpinned by author reflexivity supported our attention to the emotional landscape sitting beneath the ‘reported facts’ of diagnosis and treatment [22]. Women described the importance of having the emotional capacity *to* share. For some women, this would not have been possible early on diagnosis, but with space and time to process their diagnosis, they described being more able to share. Some expressed how personally helpful it was to talk about their experiences. We note this here as caution for future research to consider *trauma-informed approaches* to their methodology ensuring that women’s emotional safety/capacities are at the forefront.

Perhaps as a possible outcome of our methodology, a further benefit of this study was the development of a consumer advisory group—made up of willing participants from this study to advise on the future of this program of research. Arguably, careful listening and rapport building (central to our methodology) provided the necessary context for women to be interested in continuing to contribute to the research—described as ‘mutual respect’ in the Australian Framework [25].

To be inclusive, regardless of peoples’ previous experiences in/with research or their prior education, our methodology supported a women-centred approach to enable participants to tell their stories in their own words. Future research could consider capacity-building, or co-learning [35], to ensure people from diverse socio-economic and cultural backgrounds are appropriately supported to contribute and shape the agendas and processes of cancer control research, services and policy. This includes a learning for researchers about adhering to participatory principles [35] and an engagement with frameworks that are focussed on reducing power differentials and supporting partnerships [see 16].

Without claiming generalisability, which would be at odds with the scope of qualitative research [36], this analysis reminds us that, as cancer researchers, it is just as important that our research processes, as well as intended outcomes, benefit consumers to ensure consumers’ voices shape the future of cancer care. We have learned that legitimising consumers in cancer research requires methods, methodologies and research practices that pay careful attention to power, control and representation.

## Supplementary information

ESM 1

## Footnotes

1 ‘Consumers’ describes people who identify as having a living/lived experience, similar terms used in the literature include ‘patients and end users’, ‘service users’, ‘peer researchers’, ‘survivors’ and ‘expert by experience’.

2 Following Braun and Clarke’s (2006) suggestion, we use the term conceptualisation to indicate our constructionist framework guiding this work. That is, all meanings and experiences are socially constructed within a particular socio-cultural context. So, we were interested in participants’ views and understandings of research with attention to how they make meaning from their socio-cultural contexts.

3 We have chosen to use double quotation marks to quote direct speech and single quotation marks for our own analytical purposes.

4 For example, one participant who was diagnosed with vulval cancer asked her oncologist for her 5-year survival rates. The oncologist responded with a 50% chance of 5-year survival based on data for ovarian cancer. In P109’s words: “There’s nothing, there's no research. My oncologist could not give me the statistics of my five-year survival rate. I’ve been given a 50/50 survival rate, and that’s based on ovarian cancer, which is completely different.”
