# Supplementary material for: Exploring motivations for participating in research among Australian women with advanced gynaecological cancer: a qualitative study
Source: Support Care Cancer. 2023 Aug 8;31(9):511. doi: 10.1007/s00520-023-07979-x (PMC10409726; doi:10.1007/s00520-023-07979-x)
Supplement: Supplementary file 1 — ESM 1 [file 520_2023_7979_MOESM1_ESM.pdf]

Supplementary Information

Online Resource 1: Interview Guide

Title: Exploring motivations for participating in research among Australian women with advanced gynaecological cancer: A qualitative study

Journal: Supportive Care in Cancer

Authors: Wigginton B, Reeves MM, DiSipio T[1]

Corresponding author: 1. School of Public Health, The University of Queensland, Brisbane, Queensland, Australia (email: t.disipio@uq.edu.au).

General questions for interview:

- Can you tell me about your gynaecological cancer diagnosis?
- Since your diagnosis of advanced cancer, have you experienced any unmet needs? An unmet need is a need that you have not been able to satisfy.
- Since your diagnosis, have you been informed or invited to participate in any other research?
- What role, if any, do you see research playing in supporting women with advanced gynaecological cancer?
- What would make it more inviting for you to participate in a research study that followed you over a long period of time and involved multiple points of contact?
- Can I ask why you decided to participate in this research?

Anything else questions:

- What advice would you have for women in a similar situation to you?
- Is there anything else we haven’t touched on yet that you’d like to tell me about?
